# Timing of magnetic resonance imaging affects the accuracy and interobserver agreement of anterolateral ligament tears detection in anterior cruciate ligament deficient knees

**DOI:** 10.1186/s43019-020-00082-z

**Published:** 2020-11-27

**Authors:** Audrey Xinyun Han, Tien Jin Tan, Tiep Nguyen, Dave Yee Han Lee

**Affiliations:** 1 Department of Orthopedic Surgery, Changi General Hospital, Singapore, Singapore; 2 Department of Diagnostic Radiology, Changi General Hospital, Singapore, Singapore

**Keywords:** ALL, Anterolateral ligament, ACL, Anterior cruciate ligament, ACL-deficient, Anterior cruciate ligament deficient, Radiological, MRI, Tear, Visibility, Segond fracture, Diagnostic imaging

## Abstract

**Purpose:**

We aimed to identify the anterolateral ligament (ALL) tears in anterior cruciate ligament (ACL)-deficient knees using standard 1.5-Tesla magnetic resonance imaging (MRI).

**Methods:**

We included all patients who underwent primary ACL reconstruction at our center between 2012 and 2015. Exclusion criteria included patients with multiple ligament injuries, lateral collateral ligament, posterolateral corner, and infections, and patients who underwent MRI more than 2 months after their injury. All patients (*n* = 148) had ACL tears that were subsequently arthroscopically reconstructed. The magnetic resonance (MR) images of the injured knees performed within 2 months of injury were reviewed by a musculoskeletal radiologist and an orthopedic surgeon. The patients were divided into two groups. The first group of patients had MRI performed within 1 month of injury. The second group of patients had MRI performed 1–2 months after the index injury. Both assessors were blinded and the MR mages were read separately to assess the presence of ALL, presence of a tear and the location of the tear. Based on their readings, interobserver agreement (kappa statistic (K)), sensitivity, specificity, positive predictive value (PPV), negative predictive value (NPV), and accuracy were compared.

**Results:**

The ALL was identified in 100% of the patients. However, there was a discrepancy of up to 15% in the identification of tear of the ALL. In the first group in which MRI scans were performed within 1 month of injury, the ALL tear was identified by the radiologist in 92% of patients and by the surgeon in 90% of patients (Κ = 0.86). In the second group in which MRI scans were performed within 1–2 months of the injury, the ALL tear was identified by the radiologist in 78% of patients and by the surgeon in 93% of patients (K = 0.62).

**Conclusion:**

The ALL can be accurately identified on MRI, but the presence and location of ALL tear and its location cannot be reliably identified on MRI. The accuracy in identification and characterization of a tear was affected by the interval between the time of injury and the time when the MRI was performed.

**Level of evidence:**

Diagnostic, level IIIb, retrospective.

## Introduction

Claes et al. described the presence of the anterolateral ligament (ALL) consistently seen in the lateral side of the knee. The ALL was described to be an extra-articular structure with attachment from the lateral femoral condyle to the lateral meniscus and the lateral tibial plateau [1]. Subsequently, biomechanical, cadaveric, and radiological studies were conducted to verify the role and function of the ALL [2–4].

There is a large degree of variation (51–100%) in the sensitivity of magnetic resonance imaging (MRI) for identification of the ALL [5–9]. Macchi et al. and Helito et al. described the visibility of the three segments of the intact ALL in the uninjured knee, but did not examine knees with ALL tears [7, 8].

Other studies have evaluated agreement between musculoskeletal (MSK) radiologists or between MSK radiologists and orthopedic surgeons, with regards to the ALL, ALL tears, and the location of ALL tears seen on MRI. Each study varied slightly in the methodology and this must be taken into account. Taneja reported good agreement (kappa (K) = 0.70) between two MSK radiologists in identifying the presence of the ALL as a structure [9]. Kizilgovz reported good agreement (K = 0.784–1) for the visibility of the various parts of the ALL [10].

Kosy reported good agreement (K = 0.854) between two MSK radiologists in identifying ALL injuries [11]. Ferretti reported fairly good agreement (K = 0.60–0.75) between two MSK radiologists and one orthopedic surgeon in characterizing whether or not the ALL tear was complete [12]. Park also reported K values of 0.89–0.93 for agreement between two radiologists in assessing acute anterior cruciate ligament (ACL) injury in the knee [13].

There have been no studies to evaluate how the timing of MRI could affect accuracy and interobserver agreement in identifying and characterizing ALL tears. The purpose of this study was to evaluate the prevalence of ALL tear in ACL injury according to the timing of MRI and to assess the agreement between an MSK radiologist and an orthopedic surgeon in the identification of ALL tears.

The authors aimed to establish intra-observer and interobserver agreement for identifying and characterizing the ALL and ALL tears in ACL-deficient knees, using standard 1.5-Tesla MRI. The authors hypothesized that interobserver agreement for identification of ALL tears would be high. The authors also hypothesized the interobserver agreement for identification and characterization of ALL tears would decrease with increase in the duration between index injury and the MRI.

## Methods

This was a retrospective study in a cohort of patients with ACL injury. Institutional review board approval was obtained for this research. We included all patients who underwent arthroscopic ACL reconstruction with hamstring graft between 2012 and 2015 and who had preoperative MRI performed within 2 months of their injury. Patients with Segond fracture identified on radiographs and MR images were included in this study. We excluded patients with injury to the medial and/or lateral collateral ligament requiring surgical repair or reconstruction, patients with posterolateral corner injury, and patients with concomitant fractures or who underwent combined ligamentous reconstruction. Patients who underwent preoperative MRI more than 2 months after their injury were also excluded.

All MRI was performed at our center using the Magnetom Aera 1.5-Tesla (T) MRI scanner (Siemens AG, Berlin and Munich). The MRI parameters and the primary sequences used for identification of the ALL were axial and coronal proton density-weighted turbo spin echo images (Table 1).

**Table 1:** Magnetic Resonance Imaging parameters used for the study

Sequence	TE	TR	ETL	BW	Matrix	NEX	FOV	Thickness
**Axial PD**	34	3100	53	144	512 × 512	1.0	14.0	3.0
**Sagittal / Coronal PD**	34	3000	53	144	512 × 512	1.0	14.0	3.0
**Sag T2 Fat Sat**	74	4650	35	191	320 × 320	1.0	14.0	3.5

*TE* echo time (ms), *TR* repetition time (ms), *ETL* echo train length, *BW* bandwidth (Hz), *NEX* number of excitations, *FOV* field of view (cm), *Thickness* slice thickness (mm), *PD* proton-density

Our patients were stratified into two groups - ACL-deficient patients who underwent MRI within 1 month of their index injury and ACL-deficient patients who underwent MRI more than 1 month but within 2 months of their index injury. This stratification was chosen because scar tissue sets in within 3–4 weeks after injury. Therefore, MR images obtained within a month would demonstrate minimal scarring, and MR images and accuracy may be affected once scar tissue develops between 1 and 2 months after injury. The authors propose that once scarring sets in, the acutely injured structures would be more difficult to identify on MRI.

**Fig. 1:**
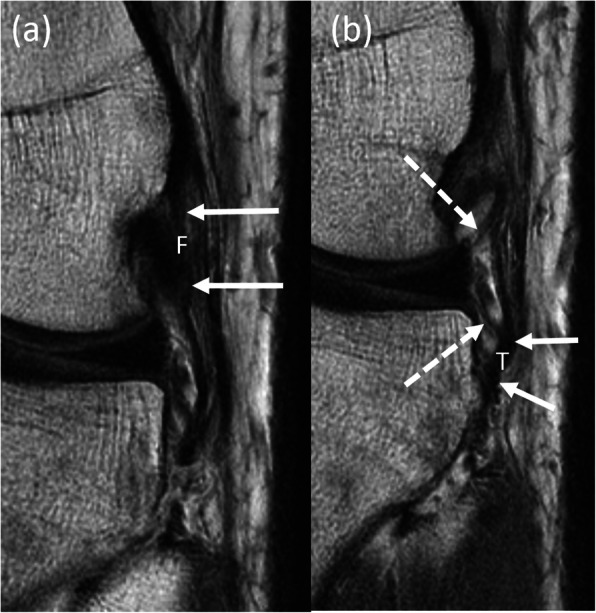
Normal radiological features of the anterolateral ligament (ALL). Serial coronal proton density weighted magnetic resonance (MR) images of the left knee in a 21-year old patient demonstrate the normal features of the femoral (**a**) (F, solid white arrows) and (**b**) meniscal (dashed white arrows) and tibial (T, solid white arrows) portions of the ALL

**Fig. 2:**
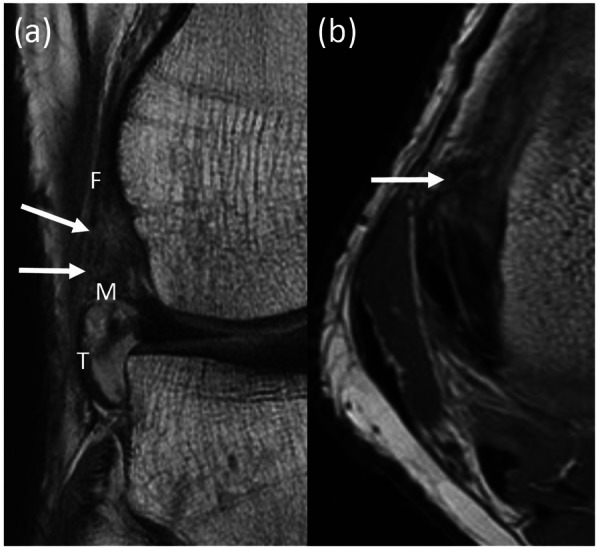
Radiological features of injuries to the femoral part of the anterolateral ligament (ALL). Coronal (**a**) and axial (**b**) proton density weighted magnetic resonance (MR) images of the right knee in a 32-year old patient demonstrate focal discontinuity, in keeping with a tear of the femoral (F, solid white arrows) portion of the ALL. The intact appearance of the meniscal (M) and tibial (T) portions is also shown

**Fig. 3:**
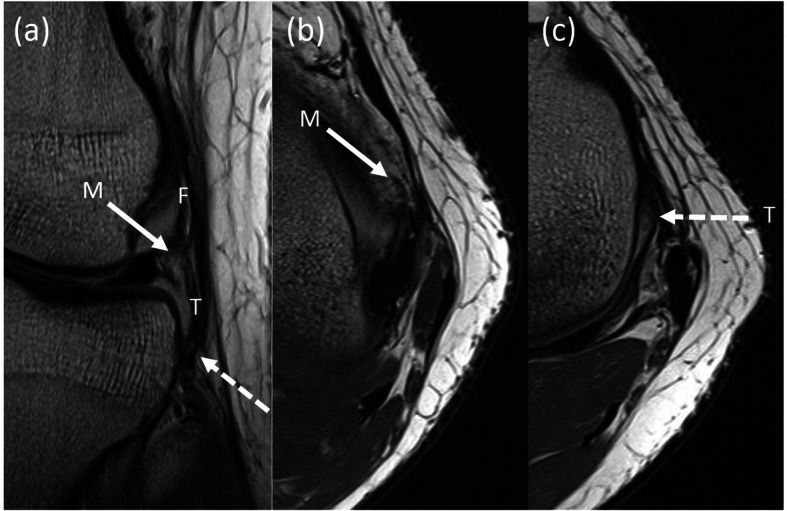
Radiological features of injury to the meniscal and tibial parts of the anterolateral ligament (ALL). Coronal (**a**) and axial (**b** and **c**) proton density magnetic resonance (MR) images of the left knee in a 22-year old patient demonstrate focal irregular attenuation, in keeping with tears of the meniscal (M, solid white arrows) and tibial (T, dashed white arrows) portions of the ALL. The femoral (F) portion is also shown and appears intact

The MR images were read twice each by a fellowship-trained MSK radiologist and an orthopedic surgeon. They were first asked to identify the ALL according to the protocol described by Helito et al. [7]. If they were able to identify the ALL on MRI, they were then asked to identify if there was a tear in the ALL. If an ALL tear was present, they were asked to identify the location of the tear (i.e. femoral, meniscal, tibial). Figure 1 shows the normal radiological features of the ALL; Fig. 2 shows the radiological features of injury to the femoral part of the ALL and Fig. 3 shows radiological features of injuries to the meniscal and tibial parts of the ALL.

Helito et al. described the ALL based on previous anatomical studies - femoral, meniscal, and tibial. The femoral part of the ALL was defined as the origin to the bifurcation point. The meniscal part of the ALL was defined as the bifurcation point to the meniscal insertion. The tibial part of the ALL was defined as the bifurcation to the tibial insertion [7].

The readings made by the MSK radiologist and the orthopedic surgeon were then compared for the calculation of intra-observer and interobserver agreement. In this study, the readings of the MSK radiologist were taken as the gold standard in view of his more extensive experience in MSK imaging. The accuracy of the orthopedic surgeon in determining if there was an ALL tear and the location of the tear on the MRI was referenced against the findings of the MSK radiologist.

## Statistical analysis

All statistical analysis was performed using IBM SPSS Statistics 21.0. The observed proportionate agreement and Cohen’s kappa coefficient were calculated to assess interobserver agreement in identifying and characterizing ALL tears in patients with ACL-deficient knees. The specificity, sensitivity, positive predictive value, and negative predictive values were also calculated to assess the accuracy of identifying and characterizing ALL tears. Accuracy is defined as the number of accurate assessments (true positives + true positives) over the number of total assessments (true positives + true negatives + false positives + false negatives).

## Results

Between 2012 and 2015, there were 368 ACL reconstructions performed at our institution. Of these, 220 were excluded because the patients had concomitant posterolateral corner injuries (*n* = 8) or did not undergo MRI within 2 months of their injury (*n* = 215). The remaining 148 patients all underwent MRI within 2 months of their injury. There were 3 patients who fell into both of these groups.

The patients were divided into 2 groups - group 1: patients who underwent MRI within a month of their injury (*n* = 88) and group 2: patients who underwent MRI more than a month but within 2 months of their index injury (*n* = 60).

### Identification of the ALL

The ALL was identified on the MRI images in all patients across the two groups. There was no intra-observer variation in the readings.

### Identification of tears in the ALL

In group 1 (*n* = 88), the radiologist identified a tear in the ALL in 81 patients (92%) and the orthopedic surgeon identified a tear in 79 patients (90%) (Table 2). The Cohen’s K coefficient was 0.86 for identification of ALL tears on MRI performed within 1 month of the index injury. In group 2 (*n* = 60), the radiologist identified a tear in the ALL in 47 patients (78%) and the orthopedic surgeon identified a tear in 50 patients (83%). The two assessors were in agreement on the assessment of 37 of the patients. The Cohen’s K coefficient was 0.62 for this group. The identification of a tear of the ALL was between 2 and 9% discrepant depending on the interval of time between the MRI and the injury. Non identification of the presence of a tear on MRI was affected by the time interval between the injury and the acquisition of MRI (*p* value <0.01).

**Table 2:** Identification of ALL tears in group 1 and group 2

Group 1 (***n*** = 88)			Group 2 (***n*** = 60)		
	Set as gold standard			Set as gold standard	
	MSK radiologist (ALL tears detected), *n*	MSK radiologist (ALL tears not detected), *n*		MSK radiologist (ALL tears detected), *n*	MSK radiologist (ALL tears not detected), *n*
Orthopedic surgeon (ALL tears detected), *n*	74	5	Orthopedic surgeon (ALL tears detected)	37	13
Orthopedic surgeon (ALL tears not detected), *n*	7	2	Orthopedic surgeon (ALL tears not detected)	10	0
Sensitivity	0.91		Sensitivity	0.79	
Specificity	0.29		Specificity	1	
PPV	0.94		PPV	0.74	
NPV	0.22		NPV	0	
Accuracy	0.86		Accuracy	0.62	
Kappa	0.86		Kappa	0.62	

Group 1 refers to patients who underwent magnetic resonance imaging (MRI) within 1 month of their index injury

Group 2 refers to patients who underwent MRI within 1–2 months of their index injury

*ALL* anterolateral ligament, *MSK* musculoskeletal, *PPV* positive predictive value, *NPV* negative predictive value

The sensitivity for identification of ALL tears was 0.91 in group 1 and 0.79 in group 2. The positive predictive value for patients in group 1 was 0.94, whereas it was 0.74 for patients in group 2. The negative predictive value for identification of ALL tears in group 1 was 0.22. Accuracy for identification of ALL tears reduced over time; the accuracy in group 1 was 0.86, but was only 0.62 in group 2.

**Fig. 4:**
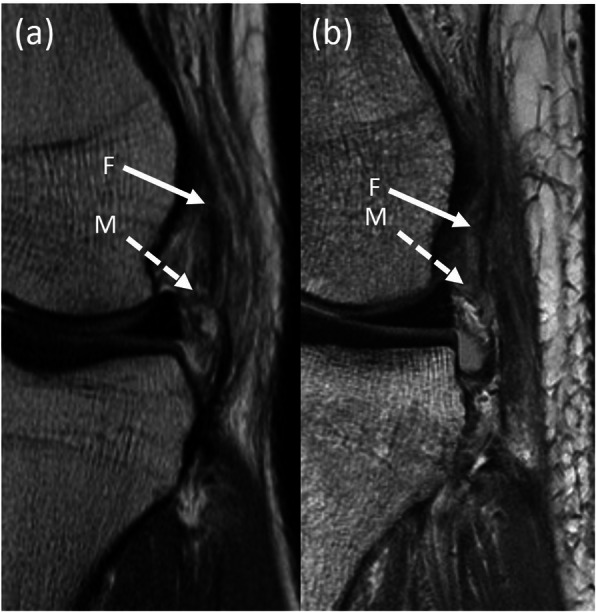
Comparison of the radiological features of anterolateral ligament (ALL) injury in group 1 and group 2. Coronal proton density magnetic resonance (MR) images of the left knee obtained less than 1 month after injury in a 22-year old patient (**a**) and obtained between 1 and 2 months after injury in a 31-year old patient (**b**). Focal tear of the femoral portion of the anterolateral ligament (F, solid white arrow) is less conspicuous in (**b**) as compared to (**a**), presumably due to scarring which has occurred by the time of imaging. Visibility of the torn meniscal portion of the anterolateral ligament (M, dashed white arrows) is also reduced in (**b**) as compared to (**a)** secondary to background scarring

As observed in Table 2, there was a trend toward lower sensitivity, positive predictive value, negative predictive value, accuracy, and the K coefficient for interobserver agreement, with increasing time interval between the index injury and the acquisition of MRI. Figure 4 shows a comparison of the radiological features of ALL injury in group 1 and group 2.

### Identifying location of the tears within the ALL

After being able to identify a tear in the ALL, the radiologist and the orthopedic surgeon were tasked to identify the location of the tear (femoral, meniscal, tibial portion of the ALL). In group 1, 60 femoral-side tears were identified by the radiologist and 41 were identified by the orthopedic surgeon (Table 3). The two readers were in agreement on 43 out of the 88 patients; the K coefficient was 0.09 and the accuracy was 0.54. The radiologist identified 67 meniscal-side tears and the orthopedic surgeon identified 63 of these. The two readers were in agreement on 64 out of 88 patients; the K coefficient was 0.30 and the accuracy was 0.73. The radiologist identified 19 tibial-side tears and the orthopedic surgeon identified 17 of these. The two readers were in agreement on 70 out of 88 of these patients; the K coefficient was 0.38 and the accuracy was 0.80.

**Table 3:** Localization of ALL tears in groups 1 and 2, based on the three portions (femoral, meniscal and tibial tears)

Femoral			Meniscal			Tibial		
	MSK radiologist (ALL tears detected), *n*	MSK radiologist (ALL tears not detected), *n*		MSK radiologist (ALL tears detected), *n*	MSK /radiologist (ALL tears not detected), *n*		MSK radiologist (ALL tears detected), *n*	MSK radiologist (ALL tears not detected), *n*
**Group 1**								
Orthopedic surgeon (ALL tears detected), *n*	30	11	Orthopedic surgeon (ALL tears detected), *n*	53	10	Orthopedic surgeon (ALL tears detected), *n*	9	8
Orthopedic surgeon (ALL tears not detected), *n*	30	17	Orthopedic surgeon (ALL tears not detected), *n*	14	11	Orthopedic surgeon (ALL tears not detected), *n*	10	61
Sensitivity	0.5		Sensitivity	0.79		Sensitivity	0.47	
Specificity	0.60		Specificity	0.48		Specificity	0.12	
PPV	0.73		PPV	0.84		PPV	0.65	
NPV	0.36		NPV	0.56		NPV	0.14	
Accuracy	0.54		Accuracy	0.73		Accuracy	0.80	
Kappa	0.09		Kappa	0.30		Kappa	0.38	
**Group 2**								
Orthopedic surgeon (ALL tears detected), *n*	27	16	Orthopedic surgeon (ALL tears detected), *n*	26	26	Orthopedic surgeon (ALL tears detected), *n*	3	25
Orthopedic surgeon (ALL tears not detected), *n*	11	6	Orthopedic surgeon (ALL tears not detected), *n*	2	6	Orthopedic surgeon (ALL tears not detected), *n*	0	32
Sensitivity	0.71		Sensitivity	0.93		Sensitivity	1	
Specificity	0.27		Specificity	0.19		Specificity	0.56	
PPV	0.63		PPV	0.50		PPV	0.11	
NPV	0.35		NPV	0.75		NPV	0	
Accuracy	0.55		Accuracy	0.53		Accuracy	0.58	
Kappa	−0.02		Kappa	0.11		Kappa	0.12	

Group 1 refers to patients who underwent magnetic resonance imaging (MRI) within 1 month of their index injury

Group 2 refers to patients who underwent MRI within 1–2 months of their index injury

*ALL* anterolateral ligament, *MSK* musculoskeletal, *PPV* positive predictive value, *NPV* negative predictive value

In group 2, the radiologist identified 38 femoral-side tears and the orthopedic surgeon identified 43 of these. They were in agreement on 33 out of 60 patients; the K coefficient for identifying femoral-side tears in the ALL on MRI between 1 and 2 months of index injury was − 0.02 and the accuracy was 0.55. The radiologist identified 28 meniscal-side tears and the orthopedic surgeon identified 52 of these. The two readers were in agreement on 32 out of 60 patients; the K coefficient was 0.11 and the accuracy was 0.53. The radiologist identified 3 tibial-side tears and the orthopedic surgeon identified 28 of these. The two readers were in agreement on 35 out of 60 patients; the observed proportionate agreement was 0.58 and the K coefficient was 0.12.

As observed in Table 3, the interobserver agreement (K coefficient) for identification of location of the tears of the ALL reduced with increasing time interval between the patient’s injury and the acquisition of MRI. The other parameters such as positive predictive value, negative predictive value, and accuracy were also noted to reduce with time, but only in the meniscal and tibial groups.

## Discussion

Based on our findings, the ALL was identified as a structure in 100% of patients with ACL-deficient knees both by the MSK radiologist and by the orthopedic surgeon. The incidence of ALL tears identified in our ACL-deficient cohort was 90% in patients who underwent MRI within 1 month of injury. The identification of ALL injuries in ACL-deficient knees was inversely proportional to the time interval between the index injury and the acquisition of MRI. Distal ALL (meniscal and tibial) injuries were more common and were identified with better accuracy and interobserver agreement as compared to proximal ALL injuries.

The available literature indicates variability in the visualization of the ALL on MRI. Claes was able to identify the ALL in 76% of his study cohort. Other authors report agreement between two radiologists for identification of the ALL as a structure in 93–100% of cases [6–8]. However, Taneja et al. identified the ALL in only 51% of their cohort [9]. In our study, the ALL was identified both by the orthopedic surgeon and by the radiologist in 100% of patients. Kizilgoz et al. identified the ALL on 82% of 206 MRI examinations performed in patients with knee injuries; 53.3% of their cohort had both ACL and concomitant ALL injury [10]. The incidence of concomitant ALL injuries was 90% in our cohort of patients with ACL-deficient knees.

The K coefficient (k = − 0.02 to 0.38) was low for the identification of tears in various parts of the ALL in our study. This is consistent with the kappa values reported by Hartigan et al. [6]. This suggests that despite being able to successfully identify the various parts of the ALL on MRI, it may not be easy to reliably identify the location of ALL tears. Claes reports that 77.8% of ALL injuries occur in the distal tibial portion [5]. Claes divided the ALL into proximal and distal portions. Proximal refers to the part of the ALL from the femoral lateral epicondyle to the meniscofemoral portion and distal refers to the part of the ALL from the meniscofemoral portion to the distal tibial insertion posterior to Gerdy’s tubercle [1]. In our study, the tears on the distal portion (meniscal and tibial tear) represented 68% of the total ALL injuries in group 1, which is similar to the Claes study.

Hartigan et al. report that agreement is poorest in identifying femoral-side ALL tears (k = 0.04–0.14) and tibial-side ALL tears (k = 0.31–0.55) [6]. Similarly, we also noted lower interobserver agreement or kappa values in identifying femoral-side tears (k = − 0.02 - 0.09) as compared to tibial-side tears (k = 0.12–0.38).

Other authors have reported better interobserver agreement in their studies. Taneja et al. and Bilfeld et al. conducted studies with a smaller sample size and assessed the agreement between two MSK radiologists [9, 14]. Ferretti et al. report better agreement (k = 0.60–0.75) among three readers - two radiologists and one orthopedic surgeon; however, they identified ALL injuries in a 26-patient cohort based on images acquired using a 3 T MRI machine [12].

In our study, it is important to take into account that we were trying to identify tears in three parts of the ALL in ACL-injured knees. We report the best interobserver agreement in identifying tibial-side tears of the ALL. This is consistent with the Macchi and the Helito studies where the best identification rates were for tears in the tibial part of the ALL [7, 8].

Porrino et al. conducted an MRI study of 53 knees and concluded that details of the ALL were difficult to discern due to the confluence of the ALL with the fibular collateral ligament at its femoral insertion. This could possibly explain the poor agreement in picking up femoral-side ALL tears [15]. Another challenge to accurately identifying ALL tibial-side injuries on MRI would be the difficulty in differentiating peripheral tears of the lateral meniscus with injury to the meniscal portion of the ALL.

Devitt et al. performed an MRI study comparing the visibility of the ALL in ACL-injured and ACL-intact knees [16]. The visibility of the ALL in three regions - femoral, meniscal, and tibial - was assessed by an MSK radiologist and an orthopedic surgeon. They reported 93% agreement and k = 0.86 for visualization of the ALL in ACL-intact knees, and 71% agreement and k = 0.52 in ACL-deficient knees. The ALL was identified more reliably in the ACL-intact knees than in the ACL-deficient knees. They reported that the visibility of the ALL was poorest at the femoral side and best at the tibial side, which is similar to our findings [16].

Devitt et al. acknowledged that they did not take into consideration the agreement for identifying such injuries in relation to the timing of the MRI [16]. In our study, we looked at the incidence and location of the ALL injury on MRI in a cohort of patients with ACL injury, taking into account the timing of the MRI. We stratified our results based on the time interval between the index injury and the acquisition of MRI. The length of this interval affected the interobserver agreement for identifying ALL tears. The discrepancy in identifying tears in the ALL when the MRI was performed within 1 month of the index injury was 2%. In the other group in whom MRI was performed after 1 month but within 2 months of the index injury, the discrepancy increased to 15% (the MSK radiologist reported ALL tears in 78% of patients, whereas the orthopedic surgeon reported ALL tears in 93% of patients). Therefore, we showed that the longer interval between the index injury and the MRI led to statistically significantly poorer accuracy/interobserver agreement for identification of ALL injuries.

Based on the guidelines proposed by Landis and Koch in 1977, the magnitude of reliability is described as follows: kappa values between 0 and 0.20 indicate slight agreement, values between 0.21 and 0.40 indicate fair agreement, values between 0.41 and 0.60 indicate moderate agreement, values between 0.61 and 0.80 indicate substantial agreement and values between 0.81 and 1.00 indicate almost perfect agreement. A negative value indicates poor agreement [17].

In our study, there “almost perfect” agreement between our two assessors in terms of localization of meniscal and tibial-sided tears in group 1. However, the agreement was only “substantial” in group 2, further affirming our previous point that the interval between the index injury and the MRI affects the agreement for identification of ALL tears in every location. It appears that the longer the interval between the index injury and the acquisition of MRI, the more disagreement there is between readers. This could be due to increased MRI scar artefact.

The strengths of our study are the following: (1) all MRI examinations in our study were performed using the same standard sequence using the 1.5 Tesla MRI machine at our center; (2) all MR images were read by two specialists (an MSK radiologist and an orthopedic surgeon); (3) we stratified our cohort based on the time interval between the index injury and the acquisition of MRI; and (4) we studies a larger sample of patients than most of the other studies published on this topic.

One limitation that we acknowledge is the use of a 1.5-T MRI machine and the 3-mm thickness of the MRI slices. Ferretti et al. report that the rate of failure to characterize the ALL is twice as high in patients who undergo 1.5-T MRI compared with 3-T MRI [18]. Patel et al. recommend a 3.0-T MRI system with a dedicated knee coil to achieve a slice thickness between 0.5 and 1.0 mm, to better define the ALL on MRI [19]. This indicates that current imaging modalities and protocols may not be sufficiently sensitive to accurately pick up ALL injuries. However, in this study, we used the MRI machines and protocols that we work with. We wanted to determine the ability of surgeons using current imaging modalities to identify ALL injuries.

Another limitation of our study was that after stratification the tear based on its sub-position on the ALL, the sample size became too small to determine statistical significance when comparing against preoperative knee outcome scores. Our data on ALL tears and the location of ALL tears also does not help us understand which tears are significant, which tears will heal, and most importantly, which tears to address surgically. Further studies will be required to address all these aspects to determine the best management of ALL injuries in the ACL-deficient knee. However, this study is a step toward understanding the imaging of the ALL. With it and with the understanding of the limitations of using MRI to diagnose ALL tears, surgeons can study these tears more closely and then choose to effect their treatment algorithm.

## Conclusion

The ALL can be accurately identified on MRI. The incidence of ALL tears as identified on MRI in a cohort of patients with ACL-deficient knees was high at 90%. Distal injuries of the ALL are more common. The time interval between the index injury and MRI is inversely proportional to the accuracy and interobserver agreement for identification of ALL injuries.

## Data Availability

Not applicable.

## Abbreviations

ACL: Anterior cruciate ligament
ALL: Anterolateral ligament
MRI: Magnetic resonance imaging
MSK: Musculoskeletal
